# The short- and long-term effects of yoga on relaxation states measured by the Smith Relaxation States Inventory 3

**DOI:** 10.1192/j.eurpsy.2024.1416

**Published:** 2024-08-27

**Authors:** T. A. Renkó, Á. Schmelowszky

**Affiliations:** ^1^Department of Psychiatry and Psychotherapy; ^2^Clinical Psychology Department, Semmelweis University; ^3^Institute of Psychology, ELTE PPK, Budapest, Hungary

## Abstract

**Introduction:**

The beneficial effects of yoga have been researched for decades, and in some countries it is also used in health care to maintain physical and mental health. Its effectiveness in the treatment of stress and anxiety, as well as in achieving a relaxed state, is supported by numerous studies.

**Objectives:**

In the present research, our aim was to investigate the direct and subclinical effects of yoga, where the subjects did at least 10 minutes of yoga a day for two weeks. Our hypotheses are that the participants experience relaxation, mindfulness and positive emotions significantly (1) more often and (2) more intensely as a result of yoga.

**Methods:**

We included 25 average population, healthy people between the ages of 18 and 30, who exercised at least 10 minutes of yoga a day for two weeks with the help of a mobile app. We used the Smith Relaxation States Inventory (SRSI3) and its disposition-measuring version (SRSI3d), which examine 19 relaxation states (R-states) presumably related to relaxation, divided into 4 categories: basic relaxation, mindfulness, positive energy and transcendence. During the statistical analyses, the values taken at the beginning of the research, before practice, were compared with the values taken directly after the last practice using the Wilcoxon test. Bonferroni correction was used to correct the first-order error that increases when testing several hypotheses simultaneously.

**Results:**

Immediately after practicing yoga, the participants had significantly higher basic relaxation (M0=2.74, M1=4.24, p<0.0001), awareness (M0=2.71, M1=2.89, p<0.0001) and positive energy (M0=3.88, M1= 4.81, p<0.0001) and in the long term they experienced significantly more relaxation (M0=3.12, M1=3.94, p<0.0001), awareness (M0=3.41, M1=4.40, p<.0001), positive energy (M0= 4.39, M1=5.14, p<0.001) and transcendence (M0=3.23, M1=4.05, p=0.001).

**Conclusions:**

Based on our results, yoga can be an effective additional tool in maintaining and improving health, but also in improving the condition and quality of life of mental and somatic patients.

**Disclosure of Interest:**

None Declared

